# Weight-shifting-based robot control system improves the weight-bearing rate and balance ability of the static standing position in hip osteoarthritis patients: a randomized controlled trial focusing on outcomes after total hip arthroplasty

**DOI:** 10.7717/peerj.15397

**Published:** 2023-05-17

**Authors:** Shigeaki Miyazaki, Go Yamako, Hideki Arakawa, Takero Sakamoto, Tsubasa Kawaguchi, Kirari Ito, Etsuo Chosa

**Affiliations:** 1 Rehabilitation Unit, University of Miyazaki Hospital, Miyazaki, Japan; 2 Department of Mechanical Engineering, Faculty of Engineering, University of Miyazaki, Miyazaki, Japan; 3 Department of Orthopaedic Surgery, Faculty of Medicine, University of Miyazaki, Miyazaki, Japan

**Keywords:** Weight-bearing rate, Balance ability, Static standing position, Hip osteoarthritis, Total hip arthroplasty, Robot exercise, Robotic rehabilitation

## Abstract

**Background:**

After a total hip arthroplasty (THA), standing and walking balance are greatly affected in the early stages of recovery, so it is important to increase the weight-bearing amount (WBA) on the operated side. Sometimes, traditional treatments may not be enough to improve WBA and weight-bearing ratio (WBR) on the operated side in a satisfactory way. To solve this problem, we came up with a new weight-shifting-based robot control system called LOCOBOT. This system can control a spherical robot on a floor by changing the center of pressure (COP) on a force-sensing board in rehabilitation after THA. The goal of this study was to find out how rehabilitation with the LOCOBOT affects the WBR and balance in a static standing position in patients with unilateral hip osteoarthritis (OA) who had a primary uncemented THA.

**Methods:**

This randomized controlled trial included 20 patients diagnosed with Kellgren–Lawrence (K–L) grade 3 or 4 hip OA on the operative side and K–L grade 0 normal hip on the nonoperative side. We used the minimization method for allocation and randomly assigned patients to either the LOCOBOT group or the control group. As a result, 10 patient seach were randomly assigned to the LOCOBOT and control groups. Both groups received 40 min of rehabilitation treatment. Out of the 40 min, the LOCOBOT group underwent treatment for 10 min with LOCOBOT. The control group performed COP-controlled exercises on a flat floor instead of using LOCOBOT for 10 of the 40 min. All theoutcome measures were performed pre-THA and 11.9 ± 1.6 days after THA (12 days after THA). The primary outcome measure included WBR in the static standing position.

**Results:**

After12 days of THA, the LOCOBOT group exhibited significantly higher mean WBR and WBA (operated side) values than the control group. Furthermore, the LOCOBOT group exhibited significantly lower mean WBA (non-operated side) and outer diameter area (ODA) values than the control group. From pre-THA to 12 days after THA, the LOCOBOT group exhibited a significant improvement in mean WBR and WBA (operated side). Moreover, the mean WBA (non-operated side) and ODA significantly decreased. From pre-THA to 12 days after THA, the control group showed a significant increase in total trajectory length and ODA.

**Conclusions:**

The most important finding of this study was that patients were able to perform the LOCOBOT exercise as early as the second day after THA, and that WBR and ODA significantly improved by the 12th day after THA. This result demonstrated that the LOCOBOT effectively improves WBR in a short period of time after THA and is a valuable system for enhancing balance ability. This expedites the acquisition of independence in activities of daily living after THA and may contribute to optimizing the effectiveness of medical care.

## Introduction

Hip osteoarthritis (OA) is a degenerative joint disease that is characterized by clinical symptoms such as, limited joint range of motion, swelling, and pain. Total hip arthroplasty (THA) is a surgical procedure performed on patients with hip OA to alleviate pain, improve function, and enhance activities of daily living (ADL). THA is useful for improving quality of life (QOL), such as gait function (Laupacis et al., 1993), sports activities (Huch et al., 2005), and cardiopulmonary function (Ries et al., 1997). Patients who have received this treatment can return to their social lives early. Studies have reported that the implant survival rate for cemented THA ranges from 80%–91% at 10–15 years, 77%–84% at 20–25 years, and 73%–78% at 30–35 years (Callaghan et al., 2009; Hernández-Vaquero, Suárez-Vazquez & Fernandez-Lombardia, 2008; Mullins et al., 2007). Furthermore, in uncemented THA, the implant survival rate was 58%–96.4% at 10–14 years and 60%–77% at 15–23 years on the acetabular si+de (Tezuka et al., 2014; Emms et al., 2010; Civinini, D’Arienzo & Innocenti 2nd, 2008) and 92%–100% at 11–15 years, 85%–100% at 15–20 years, and 95% at ≥20 years on the femoral side (Ateschrang et al., 2014; Tezuka et al., 2014; Schuh, Schraml & Hohenberger, 2009; Lombardi Jr, Berend & Mallory, 2006). Both cemented and cementless procedures have been found to be effective in achieving long-term functional durability as demonstrated in previous studies.

Rehabilitation after THA has been shown to significantly improve muscle strength, hip range of motion, and gait ability (Heiberg et al., 2012; Gilbey et al., 2003; Wang, Gilbey & Ackland, 2002). Previous studies have also reported the effectiveness of incorporating resistance training, hydrotherapy, and bicycle ergometers into the rehabilitation program after THA to improve muscle strength and physical function, and reduce pain (Giaquinto et al., 2010; Liebs et al., 2010; Suetta et al., 2004). Furthermore, accelerated perioperative care and rehabilitation intervention can significantly shorten hospital stays and improved QOL three months after surgery (Larsen et al., 2008). In particular, performing early rehabilitation following THA is crucial for enhancing the weight-bearing amount (WBA) on the operated side, improving balance and gait (Miyazaki et al., 2022; Matheis & Stöggl, 2018; Hol et al., 2010). Nonetheless, conventional treatment methods may not always yield in satisfactory results in improving balance and gait by enhancing WBA (operated side) and weight-bearing ratio (WBR). To overcome this issue, we introduced a novel weight shifting–based robot control system (LOCOBOT), which is capable of controlling a spherical robot on a floor by changing the center of pressure (COP) on a force–sensing board (Yamako et al., 2023). The LOCOBOT is an exercise robot designed to be enjoyable and motivate patients when performing balance exercise.

The purpose of the present study was to elucidate the effect of rehabilitation using the LOCOBOT on WBR and balance ability in a static standing position in patients with unilateral hip OA who underwent primary uncemented THA.

## Materials & Methods

### Study design and ethical statement

This clinical intervention study was an exploratory research using a randomized controlled trial. This study was conducted after receiving the approval of the Research Ethics Committee of the Faculty of Medicine, University of Miyazaki (Approval number I-0049). This study was conducted in accordance with the Declaration of Helsinki. The study was carried out in the Rehabilitation Unit, University of Miyazaki Hospital and registered in the Protocol Registration and Results System, https://center6.umin.ac.jp/cgi-open-bin/ctr_e/ctr_view.cgi?recptno=R000044508 (UMIN-ICDR Clinical Trial, ID: UMIN000039032). The authors provided the informed consent forms to all participants. Participants provided written consent of their own free will after the authors provided them with a thorough verbal and written explanation.

### Patient selection

Inclusion criteria were (1) patients who were diagnosed with Kellgren–Lawrence (K–L) grade 3 or 4 hip OA on the operated side and who underwent primary uncemented THA during the period from May 2021 to June 2022, (2) patients with K–L grade 0 normal hip on the non-operated side, and (3) patients who consented to participate in all evaluations prior to THA and the day prior to discharge from our institutions (post-THA day 12: 11.9 ± 1.6 days) after THA. Exclusion Criteria were (1) patients with rheumatoid arthritis, trauma, femoral head necrosis, and those who received bilateral THA or revision THA, (2) patients who were unable to participate in the evaluation 12 days after THA, (3) patients requiring load restriction of the lower extremity on the operated side after THA, and (4) patients with a post-THA rehabilitation length (days) of less than 7 days.

**Figure 1:**
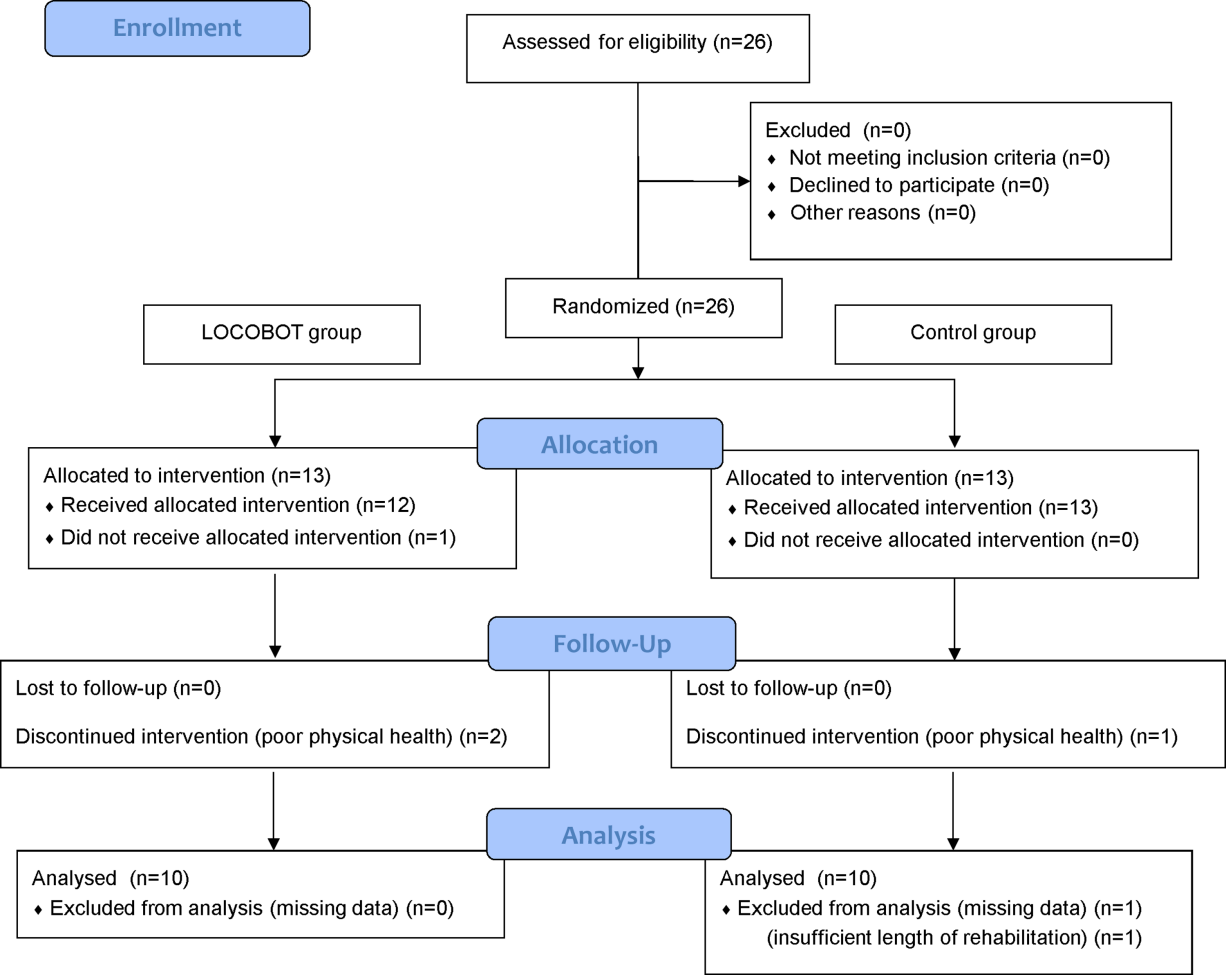
Consolidated standards of reporting trials (CONSORT) flow chart of the study showing recruitment, allocation, and analysis of subjects.

Following an assessment for eligibility, 26 patients were selected. All the patients underwent THA *via* the anterior minimally invasive surgery (AMIS) or transgluteal approach performed by four experienced orthopedic surgeons at the hospital with which the authors are affiliated. We used the minimization method for allocation, with patients randomly allocated to either the LOCOBOT treatment group (LOCOBOT group) or the control group. Allocation adjustment factors included the following: (1) gender (male/female), (2) age (≥40 years and <68 years/ ≥68 years and ≤100 years), (3) operated side (right side/left side), (4) operated side disease classification (K–L grade 3/grade 4), (5) THA approach (AMIS approach/transgluteal approach), (6) Berg Balance Scale (BBS) (≥0 points and <21 points/ ≥21 points and ≤56 points), (7) 3-m timed up & go (3-m TUG) test (≥0.01 s and <10.00 s/ ≥10.00 s and ≤119.99 s). The numerical values for (6) and (7) were used as pre-THA data. For random allocation, we used the University Hospital Medical Information Network (UMIM) Internet Data and Information System for Clinical and Epidemiological Research, Cloud version (INDICE Cloud, UMIM, Tokyo, Japan). As a result, 13 individuals were each randomly allocated to the LOCOBOT and control groups (Fig. 1). In the LOCOBOT group, one patient who required load restriction of the lower extremity on the operated side after THA did not receive the allocated intervention. Two patients who were in poor physical health discontinued intervention. In the control group, one patient discontinued intervention due to poor physical health. Two patients were excluded from analysis. One patient had missing data of primary outcome measures. The other patient was excluded because the rehabilitation length after THA was 6 days. After careful selection and strict screening, 20 patients were included in the present study (Table 1) (Fig. 1). An expert orthopedic surgeon screened the patients and an expert physical therapist guided the patients throughout the rehabilitation program.

**Table 1:** Subjects’ characteristics.

Characteristic	LOCOBOT group (*n* = 10)	Control group (*n* = 10)
Age (y)	65.3 ± 5.9	63.8 ± 6.5
Sex (male)	2	1
Height (cm)	155.0 ± 8.3	150.8 ± 8.4
Weight (kg)	55.2 ± 8.2	51.8 ± 10.9
BMI (kg/m2)	22.9 ± 2.5	22.6 ± 2.9
Length of hospitalization (day)	14.5 ± 1.4	14.6 ± 1.2
Length of rehabilitation (day)	8.6 ± 0.7	8.4 ± 1.1
Day of evaluation at discharge	11.5 ± 1.6	12.2 ± 1.6
Operated side (right)	6	5
Kellgren-Lawrence grade 4 (operated side)	7	8
THA approach (AMIS approach)	7	7

**Notes.**

BMI body mass index THA total hip arthroplasty AMIS anterior minimally invasive surgery

Age, height, weight, BMI, length of hospitalization, length of rehabilitation and day of evaluation at discharge values are means ± standard deviation.

### Weight shifting–based robot control system

**Figure 2:**
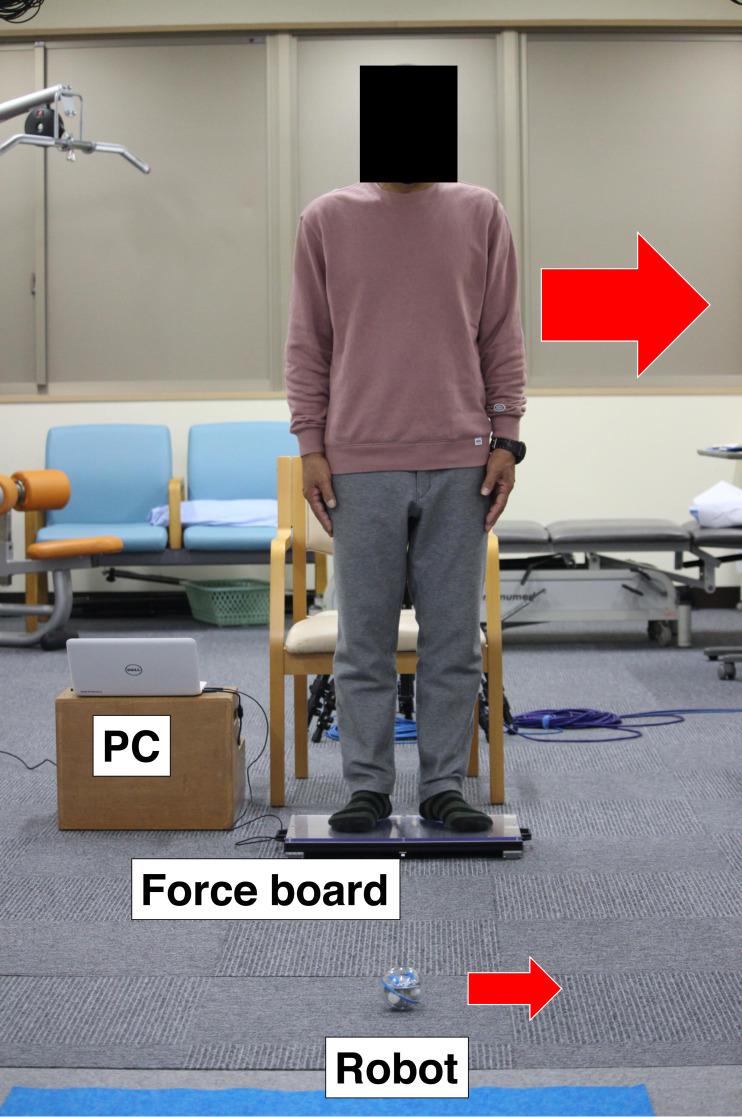
Photograph of LOCOBOT exercise. Patients stand on the force board and can move a spherical robot freely on a floor by weight shifting. The PC is connected to the force board and measures forces of the loadcell placed at each corner and calculate the center of pressure (COP) and then, send the robot the moving direction based on COP wirelessly.

LOCOBOT^®^ (LOCOBOT Inc., Miyazaki, Japan) controls a spherical robot (Sprk+, Sphero Inc., Boulder, CO, USA) based on weight-shifting board (Fig. 2). Patients can move the robot on a floor in the direction of COP based on the force board (50 × 32 cm) which has four loadcells at each corner of the board. A laptop PC is connected to the board, the vector of COP is calculated from the loads, and the moving direction is wirelessly sent to the robot. The robot stops still while the COP is within the center circle (diameter of six cm) of the board. Patients need active COP control by postural changes on the board to freely move the robot and enjoy various synchronized games, such as track races, bowling, and tag in real space, together with other users. Our previous study showed that this robot system can efficiently train the ankle joint muscles, which would improve ankle joint stability for preventing falls (Yamako et al., 2023).

### Rehabilitation protocol

The hospitalization period was 14.5 ± 1.4 days in the LOCOBOT group and 14.6 ± 1.2 days in the control group, respectively. The rehabilitation duration for the LOCOBOT and control groups were 8.6 ± 0.7, 8.4 ± 1.1 days, respectively (Table 1). Rehabilitation commenced on the day following THA, and patients were permitted to bear weight within a limited range from the day after surgery. During hospitalization, all patients received one-on-one rehabilitation for 40 min once a day. In the control group, general exercises to control the body’s COP were performed on a flat floor for 10 min during the rehabilitation. The exercises included (1) standing still and (2) shifting the COP forward and backward, left and right, diagonally forward to the right, diagonally backward to the left, diagonally forward to the left, and diagonally backward to the right. In the LOCOBOT group, treatment using the LOCOBOT was performed for 10 min of the 40-min rehabilitation session. During the rehabilitation, the patients took rest breaks depending on their fatigue levels. The rest period was excluded from the treatment time. In addition, all treatment programs were performed in a standing position involving both legs; no canes or crutches were used. The treatment program started after (1) the robot was brought to a standstill (Video S1). When the robot stands still, it indicates that the WBR of the operated and non-operated sides is equal. The goal is to achieve pelvic and trunk symmetries. (2) We implemented a program to move the robot forward and backward, left and right, diagonally forward to the right, diagonally backward to the left, diagonally forward to the left, and diagonally backward to the right, *etc*. (Video S2). If the COP shifts forward, the robot moves forward, and to move the robot, the COP must be moved in the desired direction of movement for the robot. The goal was to move the COP in all directions by moving the pelvis and trunk. (3) We created a program to knock over a toy bowling pin using LOCOBOT (Video S3). To knock over the bowling pin, the robot needed to stand still and move, which required complex and highly precise control of the COP. The LOCOBOT was used during hospitalization at the authors’ affiliated hospital, and the length of LOCOBOT usage was 7.2 ± 0.9 days.

All patients received rehabilitation with the goal of improving ADL by alleviating pain, increasing joint range of motion, acquiring normal neuromuscular coordination, strengthening gait pattern, and improving muscle strength and motor function.

### Outcome measures

This study aimed to elucidate the effects of rehabilitation using the LOCOBOT on WBR and balance ability in a static standing position. Patients with unilateral hip OA adopt a postural strategy that involves shifting the center of gravity (COG) to the non-operated side, resulting in decreased WBR (Fearon et al., 2017; Dekker, van Dijk & Veenhof, 2009). Therefore, this randomized controlled trial considered mean WBR as the most important outcome measure.

The primary outcome measure included the mean WBR (the mean load on the operated side divided by the mean load on the non-operated side) in a static standing posture (Miyazaki et al., 2021; Scoppa et al., 2013). The secondary outcome measures included mean WBA on the operated side, mean WBA on the non-operated side, total trajectory length (TTL), and outer diameter area (ODA) in a static standing posture (Miyazaki et al., 2021; Scoppa et al., 2013). The one-leg standing (OLS) test, 3-m TUG test, BBS, and functional reach (FR) test were also included. All outcome measures were performed pre-THA and 12 days after THA; no cane or crutches were used.

#### Primary outcome measures

Mean WBR. Electronic equipment was used for measuring static standing posture with two force plates (BP400600; Advanced Mechanical Technology, Inc., Watertown, MA, USA). The sampling frequency was set at 1,000 Hz. Thereafter, data was extracted by re-sampling at 100 Hz. Static standing posture was measured for 5 s using two force plates. From the measured data, we calculated the mean WBR using the analysis software (Vicon Nexus 2.12; Vicon Motion Systems, London, UK). Measurements were taken twice, and the second value was used for analysis. The instructions for quantifying posture were to (1) place the feet shoulder-width apart, (2) position the upper limbs at an angle of approximately 30° to the truck, and (3) take measurements for 5 s after confirming that the static standing posture is stable.

#### Secondary outcome measures

(1) Mean WBA (operated side), mean WBA (non-operated side), TTL, and ODA. The measurement methods and instructions were the same as those for mean WBR measurements.
(2) OLS test (Wolinsky et al., 2005; Franchignoni et al., 1998; Giorgetti, Harris & Jette, 1998). The measurements were taken with the participants’ eyes open and both hands on their hips. With 60 s set as the upper limit, measurements were taken twice for both the operated and non-operated sides. When the first measurement reached 60 s, the second measurement was stopped. The best of the two measurement results was used. Instructions to quantify movement included the following: (1) lifting one leg approximately five cm forward, (2) defining the measurement time as a maximum of 60 seconds, and (3) considering the measurement complete when the hands leave the hips.
(3) 3-m TUG test. Data were collected as previously described by Miyazaki et al. (2021). The participants were asked to stand up from a sitting position on a chair, walk to a marked spot of 3 m away, return to the chair, and sit down. The time taken for these actions was measured twice and the shorter one was adopted for analysis (Podsiadlo & Richardson, 1991). The instructions for quantifying motion were (1) place hands on the thigh while leaning lightly on the back at the starting position, (2) follow the call of the measuring person to perform a series of “maximum walking speed” actions, and (3) do the test twice and adopt the better score.
(4) BBS. To evaluate balance ability, we used a BBS consisting of 14 ADL items. Each item was given a score of 0–4 points, for a total score of 56 points. The BBS is a fall-risk assessment test; however, it can also be used to assess balance ability and overall physical function (Berg et al., 1992).
(5) FR test. The participants were instructed to extend one arm forward from a standing position, as if the body was falling forward, and to move the arm as far away from the body as possible. The maximum distance moved during that time period was measured twice, and the longest distance was used in our analysis. Instructions for quantifying movement included (1) standing with legs apart so that they do not touch, (2) lifting the arm on the wall side to 90° , and (3) extending the fingers and recording the position of the middle finger (Duncan et al., 1990).

### Statistical analysis

In the present study, we examined the effectiveness of the LOCOBOT for the WBR and balance ability in the static standing position in patients with unilateral hip OA who underwent primary uncemented THA. All data was expressed as mean ± standard deviation. The difference in each motor function parameter was examined between the two groups before THA and 12 days after THA. In the statistical analyses, parameters that followed a normal distribution were analyzed using unpaired *t*-test, whereas those that did not follow a normal distribution were analyzed using Mann–Whitney U test. Furthermore, changes in each motor function parameter in the LOCOBOT group and control group were examined before THA and at 12 days after THA, respectively. For statistical analysis, the parameters that conformed to normal distribution were analyzed using paired *t*-test, whereas those that did not conform to normal distribution were analyzed using Wilcoxon signed-rank test. All statistical analyses were performed using IBM SPSS 28.0 (released 2021; IBM Corp., Armonk, NY, USA), and a *p* value of <0.05 was considered statistically significant.

Given the lack of previous clinical studies comparing a LOCOBOT group and a control group of hip OA patients, the target number of cases was set to a total of 20 (10 each in the LOCOBOT and control group). Post-hoc power analysis was performed using “EZR” (Easy R) software version 1.61 (Saitama Medical Center, Jichi Medical University, Saitama, Japan) (Kanda, 2013). The powers of the outcome measures for detecting a significant difference with a sample size of 20 cases were 0.74 for WBR, 0.82 for mean WBA (operated side), 0.80 for mean WBA (non-operated side), and 0.88 for ODA.

## Results

Table 2 presents the difference in each parameter between the LOCOBOT and control groups before THA. For all parameters, there was no significant difference observed between the two groups. The mean WBR was 0.85 ± 0.21 in the LOCOBOT group and 0.77 ± 0.14 in the control group. The mean WBA (operated side) was 4.45 ± 0.53 N/kg in the LOCOBOT group and 4.28 ± 0.49 N/kg in the control group. Both groups had lower mean WBA on the operative side, and there was no significant difference between the two groups (mean WBR, *p* = 0.481; mean WBA on the operated side*, p* = 0.445).

**Table 2:** Comparison between LOCOBOT group and control group before THA.

	LOCOBOT group (*n* = 10)	Control group (*n* = 10)	*p*-value
Ground reaction forces: Vertical forces			
Mean WBR	0.85 ± 0.21	0.77 ± 0.14	0.481^b^
Mean WBA (operated side) (N/kg)	4.45 ± 0.53	4.28 ± 0.49	0.445^a^
Mean WBA (non-operated side) (N/kg)	5.33 ± 0.52	5.61 ± 0.46	0.225^a^
Total trajectory length	69.9 ± 23.4	57.1 ± 7.3	0.128^a^
Outer diameter area	50.9 ± 33.2	29.5 ± 18.6	0.097^a^
OLS test (operated side) (s)	34.6 ± 19.4	29.5 ± 18.0	0.554^a^
OLS test (non-operated side) (s)	41.1 ± 17.9	42.0 ± 21.4	0.631^b^
3m-TUG test (s)	8.8 ± 1.9	8.2 ± 1.2	1.000^b^
BBS	55.6 ± 1.0	55.3 ± 1.1	0.481^b^
FR test (cm)	32.9 ± 4.5	28.7 ± 6.8	0.123^a^

**Notes.**

WBA weight-bearing amount WBR weight-bearing ratio OLS one-leg standing TUG timed up & go BBS berg balance scale FR functional reach

Significantly different:^∗^*p* < .05,^∗∗^*p* < .01.

Statistical analysis:^a^ Unpaired *t*-test, ^b^ Mann-Whitney *U* test.

Table 3 presents the difference in each parameter between the LOCOBOT and control groups 12 days after THA. The mean WBR was 1.07 ± 0.13 in the LOCOBOT group and 0.85 ± 0.25 in the control group. The mean WBA (operated side) was 5.04 ± 0.31 N/kg in the LOCOBOT group and 4.41 ± 0.67 N/kg in the control group. The LOCOBOT group showed significantly higher mean WBR and mean WBA (operated side) than the control group, indicating improvement (mean WBR, *p* = 0.024; mean WBA on the operated side, *p* = 0.019). The mean WBA (non-operated side) was 4.75 ± 0.31 N/kg for the LOCOBOT group and 5.38 ± 0.69 N/kg for the control group. ODA was 25.2 ± 7.0 mm ^2^ in the LOCOBOT group and 62.2 ± 45.5 mm ^2^ in the control group. The LOCOBOT group showed significantly lower mean WBA (non-operated side) and ODA than the control group, indicating improvement (mean WBA on the non-operated side, *p* = 0.022; ODA, *p* = 0.031).

**Table 3:** Comparison between LOCOBOT group and control group at twelve days after THA.

	LOCOBOT group (*n* = 10)	Control group (*n* = 10)	*p*-value
Ground reaction forces: Vertical forces			
Mean WBR	1.07 ± 0.13	0.85 ± 0.25	0.024*^a^
Mean WBA (operated side) (N/kg)	5.04 ± 0.31	4.41 ± 0.67	0.019*^a^
Mean WBA (non-operated side) (N/kg)	4.75 ± 0.31	5.38 ± 0.69	0.022*^a^
Total trajectory length	62.4 ± 12.8	80.8 ± 25.6	0.058^a^
Outer diameter area	25.2 ± 7.0	62.2 ± 45.5	0.031*^a^
OLS test (operated side) (s)	47.1 ± 15.7	29.8 ± 21.3	0.063^b^
OLS test (non-operated side) (s)	51.0 ± 13.6	38.3 ± 21.0	0.247^b^
3m-TUG test (s)	9.6 ± 2.5	9.2 ± 2.0	0.742^a^
BBS	55.8 ± 0.4	54.6 ± 2.1	0.089^b^
FR test (cm)	33.6 ± 7.3	29.8 ± 4.9	0.315^b^

**Notes.**

WBA weight-bearing amount WBR weight-bearing ratio OLS one-leg standing TUG timed up & go BBS berg balance scale FR functional reach

Significantly different:^∗^*p* < .05,^∗∗^*p* < .01.

Statistical analysis:^a^ Unpaired *t*-test, ^b^ Mann–Whitney *U* test.

Table 4 presents the change in each parameter between measurements taken before THA and 12 days after THA in the LOCOBOT group and control group, respectively. In the LOCOBOT group, the mean WBR (0.85 ± 0.21 to 1.07 ± 0.13, *p* = 0.013) and mean WBA (operated side) (4.45 ± 0.53 to 5.04 ± 0.31, *p* = 0.004) increased significantly from before THA until 12 days after THA, indicating improvement. The mean WBA (non-operated side) (5.33 ± 0.52 to 4.75 ± 0.31, *p* = 0.004) and ODA (50.9 ± 33.2 mm ^2^ to 25.2 ± 7.0 mm ^2^, *p* = 0.041) decreased significantly from before THA till 12 days after THA, indicating improvement. In the control group, TTL (57.1 ± 7.3 mm to 80.8 ± 25.6 mm, *p* = 0.010) and ODA (29.5 ± 18.6 mm ^2^ to 62.2 ± 45.5 mm ^2^, *p* = 0.028) increased significantly from before THA till 12 days after THA, indicating deterioration.

**Table 4:** Changes in each functional parameter from before to twelve days after THA.

LOCOBOT group (*n* = 10)	Before THA	Twelve days after THA	*p*-value
Ground reaction forces: vertical forces			
Mean WBR	0.85 ± 0.21	1.07 ± 0.13	0.013*^b^
Mean WBA (operated side) (N/kg)	4.45 ± 0.53	5.04 ± 0.31	0.004**^a^
Mean WBA (non-operated side) (N/kg)	5.33 ± 0.52	4.75 ± 0.31	0.004**^a^
Total trajectory length	69.9 ± 23.4	62.4 ± 12.8	0.312^a^
Outer diameter area	50.9 ± 33.2	25.2 ± 7.0	0.041*^a^
OLS test (operated side) (s)	34.6 ± 19.4	47.1 ± 15.7	0.069^b^
OLS test (non-operated side) (s)	41.1 ± 17.9	51.0 ± 13.6	0.093^b^
3m-TUG test (s)	8.8 ± 1.9	9.6 ± 2.5	0.445^b^
BBS	55.6 ± 1.0	55.8 ± 0.4	0.705^b^
FR test (cm)	32.9 ± 4.5	33.6 ± 7.3	0.838^b^
Control group (*n* = 10)	Before THA	Twelve days after THA	*p*-value
Ground reaction forces: Vertical forces			
Mean WBR	0.77 ± 0.14	0.85 ± 0.25	0.187^a^
Mean WBA (operated side) (N/kg)	4.28 ± 0.49	4.41 ± 0.67	0.428^a^
Mean WBA (non-operated side) (N/kg)	5.61 ± 0.46	5.38 ± 0.69	0.135^a^
Total trajectory length	57.1 ± 7.3	80.8 ± 25.6	0.010*^a^
Outer diameter area	29.5 ± 18.6	62.2 ± 45.5	0.028*^a^
OLS test (operated side) (s)	29.5 ± 18.0	29.8 ± 21.3	0.953^b^
OLS test (non-operated side) (s)	42.0 ± 21.4	38.3 ± 21.0	0.612^b^
3m-TUG test (s)	8.2 ± 1.2	9.2 ± 2.0	0.147^a^
BBS	55.3 ± 1.1	54.6 ± 2.1	0.496^b^
FR test (cm)	28.7 ± 6.8	29.8 ± 4.9	0.553^b^

**Notes.**

WBA weight-bearing amount WBR weight-bearing ratio OLS one-leg standing TUG timed up & go BBS berg balance scale FR functional reach

Significantly different:^∗^*p* < .05,^∗∗^*p* < .01.

Statistical analysis:^a^ Paired *t*-test, ^b^ Wilcoxon signed-rank test.

## Discussion

In the present study, we included patients with unilateral hip OA who underwent primary uncemented THA. We evaluated WBR, WBA, TTL, and ODA in the static standing position as well as the OLS test, 3-m TUG test, BBS, and FR test before and 12 days after THA, to examine the specific effect of rehabilitation using the LOCOBOT. According to our search of the literature, there are currently no studies on the use of LOCOBOT in patients with hip OA who underwent THA. The present study is the first to examine the effect of treatment using the LOCOBOT on improving balance ability in the static standing posture, with a focus on WBR. The most important findings obtained in the present study are that the LOCOBOT can be operated from day 2 after THA and that of the mean length of rehabilitation of 8.6 days in the LOCOBOT group, the mean length of LOCOBOT usage was 7.2 days; in this short time, a significant improvement was obtained in terms of WBR and ODA. This result revealed that the LOCOBOT is a useful system that effectively improves WBR and balance ability in a short period after THA.

As a result of weight-bearing pain, patients with unilateral hip OA adopt a posture strategy of shifting the COG to the non-operated side (Fearon et al., 2017; Dekker, van Dijk & Veenhof, 2009). This postural strategy decreases the balancing ability during the standing posture (Truszczyńska et al., 2016). Reduced weight-bearing on the operated side occurs before THA and persists after THA. Consequently, an asymmetry of the trunk caused by unequal load of the legs should be corrected in the process of posture re-education after THA (Truszczyńska et al., 2014). The static standing posture WBR before THA was 0.85 in the LOCOBOT group and 0.77 in the control group, which indicates that the WBA was lower on the operated side before THA in both groups. In hip OA, movement, walking, and weight-bearing pain exacerbate as the disease progresses and becomes persistent. In the present study, all the participants had weight-bearing pain, and reduced WBA on the operated side was observed. Compared to the control group, the LOCOBOT group showed a significant higher in WBR and WBA on the operated side 12 days after THA. Furthermore, the LOCOBOT group showed a significant increase in WBR from 0.85 before THA to 1.07 twelve days after THA. In other words, WBA was greater on the non-operated side before THA, and 12 days after THA, the WBR of the operated and non-operated sides became equal, indicating that WBA increased on the operated side. Matheis & Stöggl (2018) reported that the additional mobilization and strength training targeting the muscles of the hip joint with full weight-bearing commencing from day 3 after THA resulted in improved hip range of motion and gait ability within 1 week when compared to standard physiotherapy. These results demonstrated the effectiveness of rehabilitation with full weight-bearing early after THA. Therefore, we found that treatment using the LOCOBOT improved WBR more effectively compared to standard treatment.

Several reports have examined the effectiveness and safety of early full weight-bearing after uncemented THA. Tian et al. (2017) conducted a meta-analysis and reported that early full weight-bearing in patients who received uncemented THA is safe and does not increase the onset of postoperative complications. Furthermore, Hol et al. (2010) conducted a systematic review and reported that early rehabilitation following primary uncemented THA should enable patients to promptly support their body weight within a tolerable range. The participants of the present study were allowed to support their body weight within a tolerable range from the first day after THA. Therefore, because the LOCOBOT could be used from day 2 following surgery and LOCOBOT operation was simple and safe, the treatment was effective, and a significant improvement was observed even in a short time with 7.2 days of LOCOBOT usage. Furthermore, LOCOBOT operation can be “enjoyable” because the robot feels like one’s own body, resulting in patients experiencing little subjective fatigue and a psychologically positive effect.

In the COG shift test, the balance ability is evaluated by analyzing parameters such as the distance, area, and speed of body shifts obtained by measuring the center of foot pressure in the static standing posture (Scoppa et al., 2013). The present results revealed no significant difference between the LOCOBOT and control groups before THA; however, 12 days after THA, the LOCOBOT group showed a significant improvement in ODA compared to the control group. The fact that COG shift in static standing posture improves after THA has been previously reported (Rasch, Dalén & Berg, 2010; Wykman & Goldie, 1989). Furthermore, Di Laura Frattura et al. (2022) conducted a systematic review and reported that for up to 2 weeks after THA, the balance deteriorated more than before THA. In the present study, treatment using LOCOBOT improved balancing ability in a short period, within 12 days after THA. This is the first report of a treatment that has improved balancing ability in such a short period, and is therefore clinically significant. Reinforcing symmetry, one of the components of balance, is important in improving balancing ability (Nichols, 1997). Anker et al. (2008) reported that increased weight-bearing asymmetry reduces the efficiency of hip load/unload mechanisms and increases ankle movements, thereby increasing postural instability. Based on these reports, we consider that this is attributed to an improvement in balance ability as a result of the WBA of the operated side and the non-operated side becoming equal through the treatment program using the LOCOBOT. Furthermore, changes from before THA to 12 days after THA included a significant increase in TTL and ODA in the control group but a significant decrease in ODA in the LOCOBOT group. Balance ability temporarily decreased soon after THA and then recovered normally; however, with treatment using the LOCOBOT, we found that an improvement could be obtained without a decline in balance ability after THA (Di Laura Frattura et al., 2022).

We found that treatment with the LOCOBOT effectively improved WBR and balance ability in just 12 days after THA. This is an important finding for patients who wish to resume work as soon as possible and for those who wish to return to their home as soon as possible. Furthermore, for elderly individuals, it may reduce the risk of falls and complications such as deep vein thrombosis. A topic for future study is whether treatment with the LOCOBOT can improve gait motion. To address this question, we plan to implement treatment with the LOCOBOT for 6 weeks after THA and elucidate changes in temporo-spatial parameters as well as shifts in the COG. If LOCOBOT therapy is found to be effective for improving walking ability in the early post-THA period, it will significantly shorten the time required to gain walking independence, which currently takes about 3 months after THA (Miyazaki et al., 2022). This will promote the acquisition of independence in ADL early after THA and help optimize the efficiency of medical care, both of which are clinically significant.

One limitation of the present study is that the ongoing effect of treatment with the LOCOBOT is not thoroughly examined. Evaluations in the present study were performed before THA and 12 days after THA. To obtain an accurate conclusion regarding the ongoing effect of treatment with the LOCOBOT, a larger sample size from 12 days after THA to 3 months after THA is needed. The blinding process was not observed in the study, so the device’s effect on THA may have been overestimated.

## Conclusions

Patients with unilateral hip OA who underwent primary uncemented THA showed a significant improvement in the WBR, WBA on the operated side, and ODA 12 days after THA when using LOCOBOT, compared to the control group. Furthermore, in the LOCOBOT group, the WBA on the operated side, WBR, and ODA significantly improved from before THA to 12 days after THA. These findings indicate that the LOCOBOT is an effective tool for improving WBR and balance ability in the early postoperative period following THA. The use of LOCOBOT during rehabilitation early after THA can facilitate the acquisition of independence in ADL early after THA and help to optimize the efficiency of medical care.

##  Supplemental Information

10.7717/peerj.15397/supp-1 Supplemental Information 1 The treatment program started after the robot was brought to a standstill When the robot stands still, it indicates that the WBR of the operated and non-operated sides is equal.

10.7717/peerj.15397/supp-2 Supplemental Information 2 Treatment program 1 using the LOCOBOT If the COP shifts forward, the robot moves forward, and to move the robot, the COP must be moved in the desired direction of movement for the robot.

10.7717/peerj.15397/supp-3 Supplemental Information 3 Treatment program 2 using the LOCOBOT To knock over the toy bowling pin, the robot needed to stand still and move, which required complex and highly precise control of the COP.

10.7717/peerj.15397/supp-4 Supplemental Information 4 Supplementary Tables

10.7717/peerj.15397/supp-5 Supplemental Information 5 Study protocol

10.7717/peerj.15397/supp-6 Supplemental Information 6 CONSORT checklist

## Additional Information and Declarations

### Data Availability

The following information was supplied regarding data availability: The raw measurements are available in the Supplemental Files.

### Clinical Trial Registration

The following information was supplied regarding Clinical Trial registration: UMIN000039032

## References

[ref-1] Anker LC, Weerdesteyn V, van Nes IJ, Nienhuis B, Straatman H, Geurts AC (2008). The relation between postural stability and weight distribution in healthy subjects. Gait & Posture.

[ref-2] Ateschrang A, Weise K, Weller S, Stöckle U, de Zwart P, Ochs BG (2014). Long-term results using the straight tapered femoral cementless hip stem in total hip arthroplasty: a minimum of twenty-year follow-up. The Journal of Arthroplasty.

[ref-3] Berg KO, Wood-Dauphinee SL, Williams JI, Maki B (1992). Measuring balance in the elderly: validation of an instrument. Canadian Journal of Public Health.

[ref-4] Callaghan JJ, Bracha P, Liu SS, Piyaworakhun S, Goetz DD, Johnston RC (2009). Survivorship of a Charnley total hip arthroplasty. A concise follow-up, at a minimum of thirty-five years, of previous reports. The Journal of Bone & Joint Surgery.

[ref-5] Civinini R, D’Arienzo M, Innocenti 2nd M (2008). A ten-year follow-up of the reflection cementless acetabular component. The Journal of Bone and Joint Surgery. British Volume.

[ref-6] Dekker J, van Dijk GM, Veenhof C (2009). Risk factors for functional decline in osteoarthritis of the hip or knee. Current Opinion in Rheumatology.

[ref-7] Di Laura Frattura G, Bordoni V, Feltri P, Fusco A, Candrian C, Filardo G (2022). Balance remains impaired after hip arthroplasty: a systematic review and best evidence synthesis. Diagnostics.

[ref-8] Duncan PW, Weiner DK, Chandler J, Studenski S (1990). Functional reach: a new clinical measure of balance. Journal of Gerontology.

[ref-9] Emms NW, Stockley I, Hamer AJ, Wilkinson JM (2010). Long-term outcome of a cementless, hemispherical, press-fit acetabular component: survivorship analysis and dose-response relationship to linear polyethylene wear. The Journal of bone and joint surgery. British volume..

[ref-10] Fearon A, Neeman T, Smith P, Scarvell J, Cook J (2017). Pain, not structural impairments may explain activity limitations in people with gluteal tendinopathy or hip osteoarthritis: a cross sectional study. Gait & Posture.

[ref-11] Franchignoni F, Tesio L, Martino MT, Ricupero C (1998). Reliability of four simple, quantitative tests of balance and mobility in healthy elderly females. Aging.

[ref-12] Giaquinto S, Ciotola E, Dall’armi V, Margutti F (2010). Hydrotherapy after total hip arthroplasty: a follow-up study. Archives of Gerontology and Geriatrics.

[ref-13] Gilbey HJ, Ackland TR, Wang AW, Morton AR, Trouchet T, Tapper J (2003). Exercise improves early functional recovery after total hip arthroplasty. Clinical Orthopaedics & Related Research.

[ref-14] Giorgetti MM, Harris BA, Jette A (1998). Reliability of clinical balance outcome measures in the elderly. Physiotherapy Research International.

[ref-15] Heiberg KE, Bruun-Olsen V, Ekeland A, Mengshoel AM (2012). Effect of a walking skill training program in patients who have undergone total hip arthroplasty: followup one year after surgery. Arthritis Care & Research.

[ref-16] Hernández-Vaquero D, Suárez-Vazquez A, Fernandez-Lombardia J (2008). Charnley low-friction arthroplasty of the hip. Five to 25 years survivorship in a general hospital. BMC Musculoskeletal Disorders.

[ref-17] Hol AM, van Grinsven S, Lucas C, van Susante JL, van Loon CJ (2010). Partial versus unrestricted weight bearing after an uncemented femoral stem in total hip arthroplasty: recommendation of a concise rehabilitation protocol from a systematic review of the literature. Archives of Orthopaedic and Trauma Surgery.

[ref-18] Huch K, Müller KA, Stürmer T, Brenner H, Puhl W, Günther KP (2005). Sports activities 5 years after total knee or hip arthroplasty: the Ulm Osteoarthritis Study. Annals of the Rheumatic Diseases.

[ref-19] Kanda Y (2013). Investigation of the freely available easy-to-use software ‘EZR’ for medical statistics. Bone Marrow Transplantation.

[ref-20] Larsen K, Sørensen OG, Hansen TB, Thomsen PB, Søballe K (2008). Accelerated perioperative care and rehabilitation intervention for hip and knee replacement is effective: a randomized clinical trial involving 87 patients with 3 months of follow-up. Acta Orthopaedica.

[ref-21] Laupacis A, Bourne R, Rorabeck C, Feeny D, Wong C, Tugwell P, Leslie K, Bullas R (1993). The effect of elective total hip replacement on health-related quality of life. Journal of Bone and Joint Surgery.

[ref-22] Liebs TR, Herzberg W, Rüther W, Haasters J, Russlies M, Hassenpflug J (2010). Ergometer cycling after hip or knee replacement surgery: a randomized controlled trial. Journal of Bone and Joint Surgery.

[ref-23] Lombardi Jr AV, Berend KR, Mallory TH (2006). Hydroxyapatite-coated titanium porous plasma spray tapered stem: experience at 15 to 18 years. Clinical Orthopaedics & Related Research.

[ref-24] Matheis C, Stöggl T (2018). Strength and mobilization training within the first week following total hip arthroplasty. Journal of Bodywork and Movement Therapies.

[ref-25] Miyazaki S, Tsuruta K, Yoshinaga S, Yamaguchi Y, Fujii Y, Arakawa H, Ochiai M, Kawaguchi T, Unoki A, Sakamoto T, Tajima T, Nakamura Y, Funamoto T, Hiyoshi M, Chosa E (2022). Effect of total hip arthroplasty on improving locomotive syndrome in hip disease patients: a prospective cohort study focused on total clinical decision limits stage 3. Journal of Orthopaedic Science.

[ref-26] Miyazaki S, Yoshinaga S, Tsuruta K, Hombu A, Fujii Y, Arakawa H, Sakamoto T, Chosa E (2021). Total knee arthroplasty improved locomotive syndrome in knee osteoarthritis patients: a prospective cohort study focused on total clinical decision limits stage 3. BioMed Research International. 8;.

[ref-27] Mullins MM, Norbury W, Dowell JK, Heywood-Waddington M (2007). Thirty-year results of a prospective study of Charnley total hip arthroplasty by the posterior approach. The Journal of Arthroplasty.

[ref-28] Nichols DS (1997). Balance retraining after stroke using force platform biofeedback. Physical Therapy.

[ref-29] Podsiadlo D, Richardson S (1991). The timed up & go: a test of basic functional mobility for frail elderly persons. Journal of the American Geriatrics Society.

[ref-30] Rasch A, Dalén N, Berg HE (2010). Muscle strength, gait, and balance in 20 patients with hip osteoarthritis followed for 2 years after THA. Acta Orthopaedica.

[ref-31] Ries MD, Philbin EF, Groff GD, Sheesley KA, Richman JA, Lynch Jr F (1997). Effect of total hip arthroplasty on cardiovascular fitness. The Journal of Arthroplasty.

[ref-32] Schuh A, Schraml A, Hohenberger G (2009). Long-term results of the Wagner cone prosthesis. International Orthopaedics.

[ref-33] Scoppa F, Capra R, Gallamini M, Shiffer R (2013). Clinical stabilometry standardization: basic definitions–acquisition interval–sampling frequency. Gait & Posture.

[ref-34] Suetta C, Magnusson SP, Rosted A, Aagaard P, Jakobsen AK, Larsen LH, Duus B, Kjaer M (2004). Resistance training in the early postoperative phase reduces hospitalization and leads to muscle hypertrophy in elderly hip surgery patients–a controlled, randomized study. Journal of the American Geriatrics Society.

[ref-35] Tezuka T, Inaba Y, Kobayashi N, Sato M, Mitsugi N, Saito T (2014). Long-term results of porous-coated anatomic total hip arthroplasty for patients with osteoarthritis of the hip. The Journal of Arthroplasty.

[ref-36] Tian P, Li ZJ, Xu GJ, Sun XL, Ma XL (2017). Partial versus early full weight bearing after uncemented total hip arthroplasty: a meta-analysis. Journal of Orthopaedic Surgery and Research 17;.

[ref-37] Truszczyńska A, Drzał-Grabiec J, Rąpała K, Tarnowski A, Górniak K, Białecki J (2014). Characteristics of selected parameters of body posture in patients with hip osteoarthritis. Ortopedia Traumatologia Rehabilitacja.

[ref-38] Truszczyńska A, Trzaskoma Z, Białecki J, Drzał-Grabiec J, Dadura E, Rąpała K, Tarnowski A (2016). The effect of unilateral osteoarthritis of the hip on postural balance disorders. HIP International.

[ref-39] Wang AW, Gilbey HJ, Ackland TR (2002). Perioperative exercise programs improve early return of ambulatory function after total hip arthroplasty: a randomized, controlled trial. American Journal of Physical Medicine & Rehabilitation.

[ref-40] Wolinsky FD, Miller DK, Andresen EM, Malmstrom TK, Miller JP (2005). Reproducibility of physical performance and physiologic assessments. Journal of Aging and Health.

[ref-41] Wykman A, Goldie I (1989). Postural stability after total hip replacement. International Orthopaedics.

[ref-42] Yamako G, Ito K, Muraoka T, Chosa E (2023). Leg muscle activity and joint motion during balance exercise using a newly developed weight-shifting-based robot control system. International Journal of Environmental Research and Public Health.

