# Supplementary material for: Weight-shifting-based robot control system improves the weight-bearing rate and balance ability of the static standing position in hip osteoarthritis patients: a randomized controlled trial focusing on outcomes after total hip arthroplasty
Source: PeerJ. 2023 May 17;11:e15397. doi: 10.7717/peerj.15397 (PMC10199675; doi:10.7717/peerj.15397)
Supplement: Supplemental Information 5 [file peerj-11-15397-s005.docx]

Subjects fulfill inclusion criteria

Informed consent, disadvantages, and benefits discussed

Participants allocated to either of the two groups

Control group

LOCOBOT group

Pre-intervention evaluation WBA, WBR, TTL, ODA, OLS test, 3m-TUG test, BBS, and FR test.

Pre-intervention evaluation for WBA, WBR, TTL, ODA, OLS test, 3m-TUG test, BBS, and FR test.

Intervention:

In the 40-minute rehabilitation, exercises to control the body’s COP were performed on a flat floor for 10 minutes.

Intervention:

In the 40-minute rehabilitation, treatment using the LOCOBOT was performed for 10 minutes.

Post-intervention evaluation (12 days post-THA, and 3 months post-THA) for WBA, WBR, TTL, ODA, OLS test, 3m-TUG test, BBS, and FR test.

Post-intervention evaluation (12 days post-THA, and 3 months post-THA) for WBA, WBR, TTL, ODA, OLS test, 3m-TUG test, BBS, and FR test.

Data collection

Data Analysis
